# A Review on the Sources, Structures, and Pharmacological Activities of Lucidenic Acids

**DOI:** 10.3390/molecules28041756

**Published:** 2023-02-12

**Authors:** Chengwen Zheng, Panthakarn Rangsinth, Polly H. T. Shiu, Wen Wang, Renkai Li, Jingjing Li, Yiu-Wa Kwan, George P. H. Leung

**Affiliations:** 1 Department of Pharmacology and Pharmacy, The University of Hong Kong, Hong Kong SAR, China; 2 Department of Rehabilitation Sciences, Faculty of Health and Social Sciences, Hong Kong Polytechnic University, Hong Kong SAR, China; 3 School of Biomedical Sciences, Faculty of Medicine, The Chinese University of Hong Kong, Hong Kong SAR, China

**Keywords:** *Ganoderma lucidum*, lucidenic acids, pharmacological effects

## Abstract

*Ganoderma lucidum* has long been used as a multi-purpose plant and functional food. The pharmacological properties of *G. lucidum* are primarily attributed to its polysaccharides and triterpenoids. Ganoderic and lucidenic acids are the two major triterpenoids groups in *G. lucidum*. Despite the discovery of 22 types of lucidenic acids, research on lucidenic acids is significantly less extensive compared to that on ganoderic acid. To the best of our knowledge, for the first time, in this review, we aimed to summarize the sources, contents, chemical structures, and pharmacological effects, including anti-cancer, anti-inflammatory, antioxidant, anti-viral, neuroprotective, anti-hyperlipidemic, anti-hypercholesterolemic, and anti-diabetic properties, of lucidenic acids. Studies on lucidenic acids are still preliminary and have several limitations. Therefore, more in-depth studies with optimal designs are essential for the development of lucidenic acids as medicines, functional foods, and nutraceuticals.

## 1. Introduction

Natural products are valuable sources of biologically active substances, which may serve as promising lead compounds for new drug development. Triterpenoids are one of the largest classes of natural products. Many triterpenoids possess substantial pharmacological activity and are, therefore, of interest to medicinal chemists. Triterpenoids are usually classified into the following three groups: acyclic, tetracyclic and pentacyclic, in which tetracyclic triterpenoids can be further divided into dammarane, cucurbitane, cycloartane, protostane, and lanostane types. Dammarane-type triterpenoids are mainly distributed in *Araliaceae*, *Cucurbitaceae*, *Scrophulariaceae*, and *Rhamnaceae*. Cucurbitane-type triterpenoids are mainly found in *Cucurbitaceae*; cycloartane-type triterpenoids are abundant in *Leguminosae*, *Passifloraceae*, and *Ranunculaceae*. Protostane-type triterpenoids are mainly isolated from the *Alismataceae* family, and lanostane-type triterpenoids are from fungi [1]. The tetracyclic ring system in these triterpenoids plays a critical role in their biological activities, including their anticancer [2] and antidiabetic effects [1]. Side-chain modifications of tetracyclic ring systems can affect their pharmacological properties [3,4].

**Figure 1:**
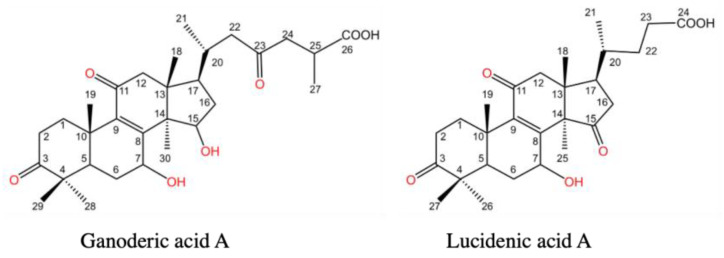
Chemical structures of ganoderic acid A and lucidenic acid A.

*Ganoderma lucidum* is a mushroom that has been used for many years as a medicinal and functional food in Far East countries to promote health and longevity. The most well-known properties of *G. lucidum* are its immunomodulatory and anti-cancer activities, which are attributed to its polysaccharides and triterpenoids [5]. Over 380 triterpenoids have been isolated from *Ganoderma* using phytochemical methods [6]. Among these triterpenoids, ganoderic acids are the most widely studied and reported. Ganoderic acids A and B were isolated from the fruiting bodies of *G. lucidum* for the first time in 1982 [7]. Ganoderic acids are C30 lanostane compounds (Figure 1). In addition to their anti-cancer and anti-diabetic effects, their anti-viral, hepatoprotective, antiplatelet, antioxidant, hypocholesterolemia, and antihistamine properties have also been reported.

Lucidenic acids, which have a C27 lanostane skeleton (Figure 1), are the second major group of triterpenoids found in the *Ganoderma* species [8]. Although some biological activities of lucidenic acids have been reported [9,10,11,12,13,14,15,16], studies that investigate their mechanisms of action and potential applications remain inadequate and preliminary. To the best of our knowledge, for the first time, in this review, we aimed to summarize the sources, contents, structures, and pharmacological activities of lucidenic acids. The findings of this review may be beneficial for the development of lucidenic acids as medicine, functional foods, and nutraceuticals.

## 2. Sources and Contents

Apart from *G. lucidum*, lucidenic acids have also been found in other *Ganoderma* species, such as *G. sinense* [17], *G. curtisii* [18], *G. colossum* [19], *G. sessile* [20], *G. tsugae* [21], *G. applanatum* [22], *G. austral* [23], *G. subresinosum* [23], and *G. hainanense* [24]. Furthermore, lucidenic acids are found in non-*Ganoderma* species [25], such as *Amauroderma rugosum* [26], *Homalium zeylanicum* [27], and potato leaves [28].

Lucidenic acids were discovered in 1984, when lucidenic acids A, B, and C were first isolated from *G. lucidum* [29]. The types and amounts of lucidenic acids in various species are listed in Table 1. *G. lucidum* is rich in lucidenic acids A, D2, and E2. The amount of lucidenic acid A in ethanol extract of *G. lucidum* fruiting bodies is 2.8 mg/g [26,30]. The amounts of lucidenic acids D2 and E2 range from 1.538 mg/g to 2.227 mg/g and 2.246 mg/g to 3.306 mg/g in grain alcohol extracts of *G. lucidum* fruiting bodies, respectively [31]. In addition to fruiting bodies, lucidenic acids can be found in other parts of *G. lucidum*, such as mycelia and spores [32]. The lucidenic acid content in fruiting bodies is higher than that in spores [33].

## 3. Chemical Structures of Lucidenic Acids

Lucidenic acids contain a tetracyclic lanostane skeleton and side chain of a carboxyl group. Lucidenic acids A, B, C, D1, D2, E1, E2, F, K, L, M, N, P and Q share the same chemical structure with the keto, hydroxyl, or acetoxy groups at C3, C7, C12, and C15 (Table 2).

Lucidenic acids G, H, I, J, O and R have structures similar to those of the aforementioned lucidenic acids, except that they have a hydroxyl substitute at C27 (Table 3). In addition, the lucidenic acid O has a distinctive carbon–carbon double-bond between C20 and C21.

The type of functional group at C3 in lanostane, number of hydroxyl groups, and type of side chain are crucial for the biological activities of triterpenoids [6,52]. For instance, the hydroxyl group at C3 is associated with α-glucosidase inhibitory activity [53]. Moreover, an increase in the number of hydroxyl groups leads to a decrease in cytotoxicity in triterpenoids [52].

## 4. Potential Pharmacological Effects of Lucidenic Acids

Lucidenic acids have potential anti-cancer, anti-inflammatory, anti-oxidant, anti-viral, anti-obesity, anti-diabetic, neuroprotective, and immunomodulatory properties (Table 4). The details are elaborated below.

### 4.1. Anti-Cancer Effect

The most widely studied pharmacological effect of lucidenic acids is their anti-cancer effect. Lucidenic acids can induce cytotoxicity in different cancer cell lines, including prostate cancer [54], leukemia [11,55,56], liver cancer [71], and lung cancer cells [43]. Lucidenic acid A decreased the viability of PC-3 prostatic cancer cells with an IC_50_ of 35.0 ± 4.1 μM [54]. Additionally, lucidenic acid A decreased the viability of HL-60 leukemia cells with an IC_50_ of 61 μM [57] and 142 μM [55] after incubation for 72 and 24 h, respectively. Furthermore, treatment with lucidenic acid A for 72 h induced cytotoxic effects in COLO205 colon cancer, HCT-116 colon cancer, and HepG2 hepatoma cells, with IC_50_ values of 154, 428, and 183 μM, respectively [57]. Both lucidenic acids A and N exhibited cytotoxicity against KB epidermal carcinoma and P388 leukemia cells [46,57]. Lucidenic acid B induced cytotoxicity in COLO205, HepG2, HL-60, and HT-29 cancer cells [57]. Among these cells, HL-60 and HepG2 cell lines were the most sensitive to lucidenic acid B, with an IC_50_ of 45.0 and 112 μM, respectively [57]. Lucidenic acid C also induced cytotoxic effects in COLO205, HepG2, and HL-60 cancer cell lines, but was not as potent as lucidenic acids A and B [57]. Lucidenic acid N also exhibited cytotoxic effects against COLO205, HepG2, and HL-60 cells, with an IC_50_ of 486, 230, and 64.5 μM, respectively [57].

The mechanism of the cytotoxic action of lucidenic acids has rarely been studied; however, lucidenic acid B has been demonstrated to induce cancer cell apoptosis via the activation of caspase-9 and caspase-3, followed by PARP cleavage [11,55]. The cytotoxic effects of lucidenic acids are also related to G1 phase cell cycle arrest [11,56]. Moreover, eukaryotic DNA polymerases can be inhibited by lucidenic acid O [49].

Apart from their direct cytotoxic effects, lucidenic acids also possess anti-proliferative properties. Lucidenic acid C exhibited moderate inhibitory activity against A549 human lung adenocarcinoma cell proliferation, with an IC_50_ between 52.6 and 84.7 μM [43]. The potential ability of lucidenic acid D to inhibit HepG2 cell proliferation has also been demonstrated based on the chemometric analysis of the spectrum–effect relationship of *Ganoderma* extracts [66].

In addition to their cytotoxic and anti-proliferative effects, lucidenic acids can inhibit cancer cell invasion, implying that they may have a potential anti-metastatic effect. For instance, 24 h incubation with 50 µM of lucidenic acids A, B, C, and N inhibited HepG2 cell invasion without affecting cell viability [58]. The mechanism of action of this anti-invasive effect remains unknown, but it may be associated with the inhibition of matrix metallopeptidase 9 (MMP-9). Lucidenic acid B has been reported to reverse phorbol myristate acetate-induced MMP-9 activity in a dose-response manner [12]. This effect is related to the suppression of both MAPK/ERK1/2 phosphorylation and IκBα protein activation while enhancing the expression of IκBα protein, leading to a decrease in NF-κB DNA-binding activity [12].

Another promising property of lucidenic acids is that certain lucidenic acids, such as lucidenic acids A, E, and N, may potentiate the anti-cancer effect of doxorubicin [59]. This synergistic effect may be beneficial, as it may lower the dosage required, and hence reduce the adverse drug reactions, such as cardiotoxicity, of doxorubicin. Lucidenic acids are considered to be safe because their cytotoxic and antiproliferative effects are specific to cancer cells. A study showed that lucidenic acid killed 50% of HL-60 leukemia cells at concentrations ranging from 19.3 to 64.5 μM and had no significant effect on the viability of normal peripheral blood lymphocytes [11].

The target binding sites of lucidenic acids in cancer cells remain unidentified. Computational molecular docking models have demonstrated promising binding energies of lucidenic acids for the Mdm2 receptor (predicted hydrogen bonding with Val93, Ile19, Gln24, Gln18 and His96) and zinc finger 439 protein (predicted hydrogen bonding with at Ser86), suggesting that they may be the target sites of lucidenic acids in breast cancer [72,73]. Mdm2 is a potent inhibitor of the p53 family of transcription factors and tumor suppressors. The function of the zinc finger 439 protein remains unknown, but it is suggested to be involved in the regulation of gene transcription. Moreover, lucidenic acids may act as potential quadruplex stabilizing ligands and promising inhibitors of Bcl-2 [74,75], which is a well-known apoptosis suppressor.

### 4.2. Anti-Inflammatory Effect

Inflammation is involved in infectious diseases and chronic disorders, such as arthritis, inflammatory bowel disease, and dermatitis. The anti-inflammatory functions of lucidenic acids have been demonstrated by a previous study, which reported that *G. lucidum* extracts containing lucidenic acids B, D1, D2, E1, and L attenuated lipopolysaccharide-induced pro-inflammatory cytokine and nitric oxide release and increased the expression levels of inducible nitric oxide synthase and cyclo-oxygenase-2 in RAW264.7 cells [65]. Similarly, lucidenic acid R suppressed 20% of nitric oxide production in lipopolysaccharide-stimulated RAW264.7 cells [51]. Moreover, an in vitro study using a protein denaturation assay demonstrated that lucidenic acid A inhibited inflammation, with an IC_50_ of 13 μg/mL [27].

In vivo anti-inflammatory effects of lucidenic acids have also been reported. In a mouse model of 12-O-tetradecanoylphorbol-13-acetate-induced ear skin inflammation, the tropical treatment of lucidenic acids A, D2, E2, and P inhibited skin inflammation with ID_50_ values of 0.07, 0.11, 0.11, and 0.29 mg/ear, respectively [60].

### 4.3. Antioxidant Effect

The thiobarbituric acid reactive substances assay has demonstrated that *G. lucidum* extract can suppress oxidative stress in rat liver mitochondria [16]. Among the different fractions of *G. lucidum* extract, the fraction with ganoderic acids A, B, C, and D, lucidenic acid B, and ganodermanontriol as major components had the highest protective effect against lipid peroxidation [16]. Nevertheless, further studies are required to confirm the antioxidant effect of lucidenic acids.

### 4.4. Anti-Viral Effect

The Epstein–Barr virus is a key risk factor for many malignant diseases, such as nasopharyngeal carcinoma and Burkitt lymphoma. Notably, lucidenic acid A, C, D2, E2, F, and P, methyl lucidenate A, methyl lucidenate E2, methyl lucidenate Q, and 20-hydroxylucidenic acid N inhibited the activation of the Epstein–Barr virus early antigen in Raji cells [50,60]. Human angiotensin-converting enzyme (hACE2) is the key receptor for the entry of severe acute respiratory syndrome coronavirus 2 (SARS-CoV-2) into target cells [76]. While the efficacy of anti-viral medications decreased with the appearance of new SARS-CoV-2 variants [10], blocking hACE2 may be an effective method to prevent SARS-CoV-2 infection [10]. The molecular docking results showed that lucidenic acid A has good binding stability to hACE2 (interaction with the amino acid residues Gln96, Asn33 and Lys26) [61]. In vitro fluorescence resonance energy transfer tests also demonstrated that lucidenic acid A inhibited hACE2 with an IC_50_ of 2 μmol/mL [61]. This suggests that lucidenic acids may be useful for the prevention or treatment of COVID-19.

In addition, molecular docking has demonstrated that lucidenic acids A, B, C, and N can bind to matrix metalloproteinase, so their effects on inhibiting the invasion of hepatitis B virus have been proposed [62]. Moreover, lucidenic acids may have potential effects on the human immunodeficiency virus (HIV). Lucidenic acid O has been reported to inhibit HIV reverse transcriptase with an IC_50_ of 67 μM [49]. Moreover, 20-hydroxylucidenic acid N and 20(21)-dehydrolucidenic acid N, which are derivatives of lucidenic acids, exhibited anti-HIV-1 protease activity [9].

### 4.5. Neuroprotective Effect

Neurodegenerative diseases have become prevalent, owing to the aging population, affecting more than 55 million people worldwide [77]. *G. lucidum* extract that contains lucidenic acids exhibited neuroprotective effects [13]. Lucidenic acids A and N and methyl lucidenic E2 inhibited acetylcholinesterase with IC_50_ values of 24.04 ± 3.46, 25.91 ± 0.89, and 17.14 ± 2.88 μM, respectively [15]. Furthermore, another study reported that lucidenic acid A inhibited acetylcholinesterase, with an IC_50_ of 54.5 μM [78]. In addition, lucidenic acid N inhibited butyrylcholinesterase activity, with an IC_50_ of 188.36 ± 3.05 μM [15]. Cholinergic neurotransmitters decline in the brains of patients with Alzheimer’s disease. The inhibition of cholinesterase by lucidenic acid may increase acetylcholine levels in the central nervous system, thus enhancing cholinergic transmission [79].

### 4.6. Anti-Hyperlipidemic Effect

Lucidenic acids have the potential to treat hyperlipidemia. Lucidenic acid N at a concentration of 80 μM reduced triglyceride accumulation in 3T3-L1 preadipocytes by approximately 30% [68]. Lucidenic acid N, methyl lucidenate E2, and methyl lucidenate F have been reported to inhibit adipocyte differentiation [69]. Butyl lucidenate N, a lucidenic acid derivative, inhibited adipogenesis in 3T3-L1 cells by downregulating the gene expression of sterol regulatory element-binding protein-1c, fatty acid synthase, and acetyl-CoA carboxylase [70]. Furthermore, lucidenic aid A has been proposed as a component that is associated with the anti-hyperlipidemic effect of Fu-Ling-Pi, a traditional Chinese medicine [63].

### 4.7. Anti-Hypercholesterolemic Effect

β-Hydroxyβ-methylglutaryl-CoA (HMG-CoA) reductase inhibitors are commonly used as lipid-lowering medications. They can reduce cholesterol biosynthesis and regulate lipid metabolism, thus preventing the incidence of mortality in coronary patients [80]. The results of virtual screening and in silico profiling have demonstrated the potential of lucidenic acids to interact with HMG-CoA reductase [67]. Additionally, another study has shown that lucidenic acid E can inhibit HMG-CoA reductase, with an IC_50_ of 42.9 ± 0.9 μM [43].

### 4.8. Anti-Hyperglycemic Effect

A study reported that lucidenic acids E, H, and Q had promising anti-hyperglycemic properties [43]. Among these, lucidenic acids E and Q inhibited α-glucosidase, with an IC_50_ of 32.5 and 60.1 μM, respectively [43]. They could also inhibit maltase, with an IC_50_ of 16.9 and 51 μM, respectively [43]. Moreover, lucidenic acid Q showed inhibitory activity against sucrase in rats, with an IC_50_ of 69.1 μM [43]. PTP1B inhibitors are promising therapeutic agents for diabetes [81]. Lucidenic acids H and E exhibited inhibitory activity against PTP1B within a concentration range of 7.6–41.9 μM [43]. In addition, lucidenic acid Q inhibited aldose reductase, which may be useful for the prevention of diabetic complications, such as neuropathy [43].

### 4.9. Other Pharmacological Effects

Apart from the aforementioned pharmacological effects, lucidenic acid I, methyl lucidenate E2, and dehydrolucidenic acid N have immunomodulatory activities that enhance recovery from neutropenia, macrophage formation, and macrophage phagocytosis [14]. In addition, a study has demonstrated that a *G. lucidum* nanogel, which contains 6.3% lucidenic acid A and 7.3% lucidenic acid H, is effective for the topical treatment of frostbite [64].

## 5. Conclusions

This review summarizes the sources, contents, chemical structures, and pharmacological effects of lucidenic acids. Lucidenic acids are a group of tetracyclic triterpenoids that possess anti-cancer, anti-inflammatory, antioxidant, anti-viral, anti-hyperlipidemic, anti-hyperglycemic, neuroprotective, and immunomodulatory properties. Previous studies on lucidenic acids are preliminary and have several limitations. Therefore, further studies are warranted for the development of lucidenic acids as medicines, functional foods, and nutraceuticals.

## 6. Future Directions

As lucidenic acids have promising pharmacological effects and different *Ganoderma* species contain different compositions of lucidenic acids, it has been proposed that the types and levels of lucidenic acids in *Ganoderma* products may serve as an indicator for quality control [82], similar to that used for ganoderic acids. Lucidenic acids and ganoderic acids are C27 and C30 lanostane triterpenoids, respectively. Theoretically, this 3-carbon difference may affect their physicochemical properties (e.g., stability and solubility), pharmacokinetic properties, and receptor binding. It is not known whether lucidenic acids are better drug candidates when compared with ganoderic acids. However, we cannot exclude the possibility that lucidenic acid may have certain pharmacological effects that ganoderic acids do not have, such as the blocking effect of lucidenic acids on hACE2, which has never been reported for ganoderic acid. Nonetheless, using lucidenic acids for the treatment or prevention of any disease cannot be proposed yet because the research findings are preliminary and inadequate. Therefore, further studies are required.

First, some effects of lucidenic acids were predicted using molecular docking. A typical example is the proposed inhibitory effect of lucidenic acid on hACE2. Further in vivo and in vitro studies are needed to verify the usefulness of lucidenic acids in the treatment of COVID-19. Similarly, the potential anti-hyperlipidemic, anti-diabetic, and neuroprotective effects of lucidenic acids were primarily studied using biochemical assays. Biological studies using in vitro, ex vivo, or in vivo models should be performed. In addition, the anti-cancer effects of lucidenic acids have been mostly demonstrated in in vitro models. As lucidenic acids exhibit low toxicity against normal cells, in vivo studies, such as in xenograft mouse models, should be considered in the future.

Second, the entire range of lucidenic acids should be studied to obtain a full picture of their structure–activity relationship. For instance, many pharmacological studies have been performed on lucidenic acids A, B, C, and N. Studies on their structures revealed that these lucidenic acids possess a hydroxyl group at the C7 position and a keto group at the C15 position. To confirm whether the hydroxyl and keto groups are essential for their pharmacological effects (e.g., cytotoxicity), the other lucidenic acids should also be studied (at least lucidenic acids E1, H, and P should be evaluated because they also contain these two functional groups).

Third, the pharmacokinetics and bioavailability of lucidenic acids have not yet been investigated. These data are crucial for drug development, especially for formulation design and dosage regimens. A pharmacokinetic study in a rat model showed that the oral bioavailability of ganoderic acid A was as low as 8.68% [83]. The bioavailability of lucidenic acids, which have chemical structures similar to those of ganoderic acids, may not be high. Nevertheless, even though lucidenic acids may not be easily absorbed in the gastrointestinal tract, lucidenic acid can still be orally active if its potency is high enough. Furthermore, the interactions between lucidenic acids and the gut microbiota should also be taken into consideration. Recent studies have reported that ganoderic acids have the potential to alleviate lipid metabolic disorders and diabetes mellitus, and ameliorate the imbalance of gut microflora in hyperlipidemic and diabetic mice [84,85]. In addition, *G. lucidum* extracts fermented by probiotics, such as *Bifidobacterium bifidum* and *Lactobacillus sakei*, could be useful to enhance learning memory and cognitive function [86] and improve immunity [87]. Probiotic fermentation of *G. lucidum* extracts induces structural changes in the ganoderic acid components. Further studies are required to investigate whether lucidenic acids can also be biotransformed into substances that will be beneficial for health.

The advantages of lucidenic acids are their versatile pharmacological effects, especially on cancer, inflammation, neuroprotection, hyperlipidemia and hypercholesterolemia. These diseases are common problems worldwide because of the aging population and unhealthy lifestyle of the general population. Lucidenic acids are mainly found in edible fungi such as *G. lucidum,* so they should be reasonably safe and can be tolerated by humans. The cultivation of *Ganoderma* fungi can provide an adequate supply of lucidenic acids. The associated production cost may even be lowered if lucidenic acids can be obtained from mycelial cultures grown in large-scale fermentations. Nonetheless, the possible disadvantages should not be neglected. For instance, the content of lucidenic acids from *Ganoderma* fungi may be varied by environmental factors, so quality control is important. In addition, different lucidenic acids may have different pharmacological effects. The isolation of a specific type of lucidenic acid from crude extracts of *Ganoderma* fungi may be difficult and costly. Lucidenic acids may have a broad spectrum of therapeutic properties but lack specific molecular targets, which may cause unwanted side effects. Therefore, much more research must be conducted to develop lucidenic acids into medicines, functional food, or nutraceuticals. It is hoped that this review can provide some insights into this research area.

## Figures and Tables

**Table 1:** The sources, molecule formulae, and amounts of lucidenic acids.

Serial Number	Lucidenic Acid Type	Molecular Formula	Species	Extraction Method	Amount	References
1	Lucidenic acid A	C_27_H_38_O_6_	*Ganoderma lucidum (*fruiting bodies)	100% Ethanol	2.8 mg/g dry weight	[30]
			*Ganoderma lucidum (*fruiting bodies)	95% Ethanol	1.53–1.74 mg/g dry weight	[34]
			*Ganoderma lucidum (*fruiting bodies)	45% Grain alcohol and chloroform	1.226–2.497 mg/g in lyophilized sample	[29,31,35]
			*Ganoderma lucidum (*fruiting bodies)	Water (soaked in 100% ethanol overnight prior to extraction)	0.4 mg/g dry weight	[36]
			*Ganoderma lucidum (*fruiting bodies)	Water	51 μg/g dry weight	[26]
			*Ganoderma lucidum* (spores)	Methanol	*	[14]
			*Ganoderma lucidum* (spores)	Supercritical fluid carbon dioxide	0.3 mg/g in extract	[37]
			Wall-removed *Ganoderma lucidum* (spores)	Water, alcohol, or a combination of the two	0.05%	[38]
			*Ganoderma hainanense* (fruiting bodies)	95% Ethanol	*	[6,24]
			*Ganoderma sinense (*fruiting bodies)	95% Ethanol	*	[17]
			*Ganoderma curtisii* (fruiting bodies)	Methanol	*	[18]
			*Ganoderma colossum (*fruiting bodies)	100% Ethanol	16 μg/mL in extract	[19]
			*Ganoderma sessile* (fruiting bodies)	80% Ethanol	*	[20]
			*Amauroderma rugosu*m (fruiting bodies)	Water	15.69 μg/g dry weight	[26]
			*Homalium zeylanicum* (barks)	70% Hydro-alcohol	*	[27]
2	Lucidenic acid B	C_27_H_38_O_7_	*Ganoderma lucidum* (fruiting bodies)	Chloroform	*	[6,35,39]
			*Ganoderma lucidum* (spores)	Methanol	*	[14]
			*Ganoderma lucidum* (spores)	Supercritical fluid carbon dioxide	72 ± 0.95 μg/g in extract	[37]
3	Lucidenic acid C	C_27_H_40_O_7_	*Ganoderma lucidum (*fruiting bodies)	Chloroform	*	[6,35,39]
			*Ganoderma lucidum* (spores)	Methanol	*	[14]
			*Ganoderma colossum* (fruiting bodies)	100% Ethanol	6.7 μg/mL in extract	[19]
			*Ganoderma sessile* (fruiting bodies)	80% Ethanol	*	[20]
			*Ganoderma tsugae (*fruiting bodies)	95% Ethanol	*	[21]
4	Lucidenic acid D1	C_27_H_34_O_7_	*Ganoderma lucidum* (fruiting bodies)	Chloroform	*	[6,35]
5	Lucidenic acid D2	C_29_H_38_O_8_	*Ganoderma lucidum (*fruiting bodies)	45% Grain alcohol and chloroform	1.538–2.227 mg/g in lyophilized sample	[31,35,40]
			*Ganoderma lucidum* (spores)	Methanol	*	[14]
			*Ganoderma sinense* (fruiting bodies)	Chloroform	*	[6,9]
			Potato leaf	Methanol: Water (4:1, *v*/*v*)	*	[28]
6	Lucidenic acid E1	C_27_H_38_O_7_	*Ganoderma lucidum (*fruiting bodies)	Chloroform	*	[35]
7	Lucidenic acid E2	C_29_H_40_O_8_	*Ganoderma lucidum (*fruiting bodies)	Methanol	0.319–1.766 mg/g dry weight (wild samples); 0.258–0.481 mg/g dry weight (cultivated samples)	[23,39,40]
			*Ganoderma lucidum (*fruiting bodies)	45% Grain alcohol	2.246–3.306 mg/g in lyophilized sample	[31]
			*Ganoderma lucidum* (spores)	Methanol	*	[14]
			*Ganoderma australe (*fruiting bodies)	Methanol	121.65 ± 4.50 μg/g dry weight	[23,39,40]
			*Ganoderma colossum (*fruiting bodies)	Methanol	201.92 ± 2.45 μg/g dry weight	[23,39,40]
8	Lucidenic acid F	C_27_H_36_O_6_	*Ganoderma lucidum* (fruiting bodies)	Ether	*	[6,39,40,41]
			*Ganoderma lucidum* (spores)	Methanol	*	[14]
			*Ganoderma curtisii (*fruiting bodies)	Methanol	*	[18]
			Potato leaf	Methanol: water (4:1, *v*/*v*)	*	[28]
			metabolites of rice	Methanol: water (4:1, *v*/*v*)	*	[25]
9	Lucidenic acid G	C_27_H_40_O_7_	*Ganoderma lucidum (*fruiting bodies)	Ethanol	*	[6,42]
			*Ganoderma lucidum* (spores)	Methanol	*	[14]
10	Lucidenic acid H	C_27_H_40_O_7_	*Ganoderma lucidum* (fruiting bodies)	Ethanol and crystallized from fraction CHCl_3_-MeOH, 9:1	*	[43,44]
11	Lucidenic acid I	C_27_H_38_O_7_	*Ganoderma lucidum* (fruiting bodies)	Ethanol and crystallized from fraction CHCl_3_-MeOH, 9:1	*	[6,44]
			*Ganoderma lucidum* (spores)	Methanol	*	[14]
12	Lucidenic acid J	C_27_H_38_O_8_	*Ganoderma lucidum* (fruiting bodies)	Ethanol and crystallized from fraction CHCl_3_-MeOH, 9:1	*	[6,44]
			*Ganoderma lucidum* (spores)	Methanol	*	[14]
13	Lucidenic acid K	C_27_H_40_O_7_	*Ganoderma lucidum* (fruiting bodies)	100% Ethanol	*	[6,44]
			*Ganoderma lucidum* (spores)	Methanol	*	[14]
14	Lucidenic acid L	C_27_H_38_O_7_	*Ganoderma lucidum* (fruiting bodies)	100% Ethanol	*	[6,44]
15	Lucidenic acid M	C_27_H_42_O_6_	*Ganoderma lucidum* (fruiting bodies)	100% Ethanol	*	[6,44]
			*Ganoderma lucidum* (spores)	Methanol	*	[14]
16	Lucidenic acid N (lucidenic acid SP1, LM1)	C_27_H_40_O_6_	*Ganoderma lucidum* (fruiting bodies)	Methanol	257.80–884.05 μg/g dry weight (wild samples); 52.53–139.08 μg/g dry weight (cultivated samples)	[23,39,45,46,47]
			*Ganoderma lucidum* (fruiting bodies)	45% Grain alcohol	0.866–2.004 mg/g in lyophilized sample	[31]
			*Ganoderma lucidum* (spores)	Methanol	*	[14]
			*Ganoderma lucidum* (spores)	Supercritical fluid carbon dioxide	161 ± 2.21 μg/g in extract	[37]
			*Ganoderma lucidum* (mycelia)	96% Ethanol	0.23–0.33 mg/g dry weight	[48]
			*Ganoderma curtisii* (fruiting bodies)	Methanol	*	[18]
			*Ganoderma sessile* (fruiting bodies)	80% Ethanol	*	[20]
			*Ganoderma tsugae* (fruiting bodies)	95% Ethanol	*	[21]
			*Ganoderma subresinosum* (fruiting bodies)	Methanol	57.50 ± 0.65 μg/g dry weight	[23,39,45,46,47]
			*Ganoderma colossum* (fruiting bodies)	Methanol	207.73 ± 2.05 μg/g dry weight	[23,39,45,46,47]
			*Ganoderma australe* (fruiting bodies)	Methanol	63.13 ± 1.45 μg/g dry weight	[23,39,45,46,47]
			*Ganoderma hainanense* (fruiting bodies)	95% Ethanol	*	[24]
17	Lucidenic acid O	C_27_H_40_O_7_	*Ganoderma lucidum* (fruiting bodies)	Acetone	*	[6,49]
18	Lucidenic acid P	C_29_H_42_O_8_	*Ganoderma lucidum* (fruiting bodies)	Methanol	*	[6,50]
			*Ganoderma lucidum* (spores)	Methanol	*	[14]
19	Lucidenic acid Q	C_27_H_40_O_6_	*Ganoderma lucidum* (fruiting bodies)	Ethyl acetate	*	[43]
			*Ganoderma lucidum* (spores)	Methanol	*	[14]
20	Lucidenic acid R	C_29_H_40_O_9_	*Ganoderma lucidum* (fruiting bodies)	80% Ethanol	*	[51]

* Not specified in the literature.

**Table 2:** Chemical structures of lucidenic acids A, B, C, D1, D2, E1, E2, F, K, L, M, N, P and Q.

Basic Chemical Structure	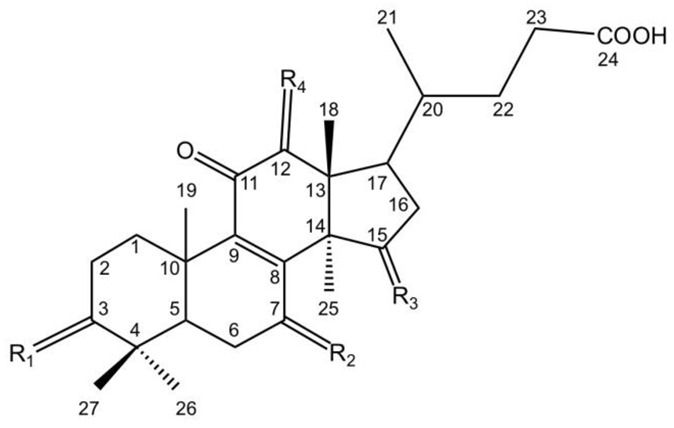				
Lucidenic Acid Type	R_1_	R_2_	R_3_	R_4_	References
Lucidenic acid A	R_1_ = O	R_2_ = -OH	R_3_ = O	R_4_ = H	[29]
Lucidenic acid B	R_1_ = O	R_2_ = -OH	R_3_ = O	R_4_ = -OH	[29]
Lucidenic acid C	R_1_ = -OH	R_2_ = -OH	R_3_ = O	R_4_ = -OH	[29]
Lucidenic acid D1	R_1_ = O	R_2_ = O	R_3_ = O	R_4_ = O	[35]
Lucidenic acid D2	R_1_ = O	R_2_ = O	R_3_ = O	R_4_ = OCOCH_3_	[40]
Lucidenic acid E1	R_1_ = O	R_2_ = -OH	R_3_ = O	R_2_ = -OH	[35]
Lucidenic acid E2	R_1_ = -OH	R_2_ = O	R_3_ = O	R_4_ = OCOCH_3_	[40]
Lucidenic acid F	R_1_ = O	R_2_ = O	R_3_ = O	R_4_ = H	[40]
Lucidenic acid K	R_1_ = O	R_2_ = O	R_3_ = O	R_4_ = -OH	[44]
Lucidenic acid L	R_1_ = -OH	R_2_ = O	R_3_ = O	R_4_ = -OH	[44]
Lucidenic acid M	R_1_ = -OH	R_2_ = -OH	R_3_ = -OH	R_4_ = H	[44]
Lucidenic acid N	R_1_ = -OH	R_2_ = -OH	R_3_ = O	R_4_ = H	[46]
Lucidenic acid P	R_1_ = -OH	R_2_ = -OH	R_3_ = O	R_4_ = OCOCH_3_	[50]
Lucidenic acid Q	R_1_ = O	R_2_ = -OH	R_3_ = -OH	R_4_ = H	[43]

**Table 3:** Chemical structures of lucidenic acids G, H, I, J, O and R.

Basic Chemical Structure	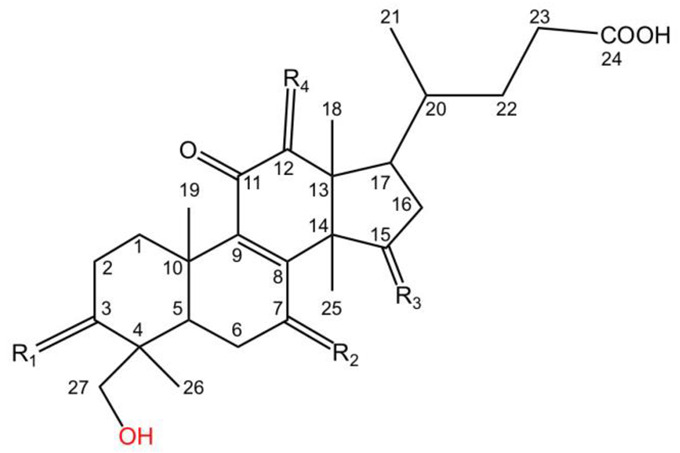				
Lucidenic Acid Type	R_1_	R_2_	R_3_	R_4_	References
Lucidenic acid G	R_1_ = O	R_2_ = -OH	R_3_ = -OH	R_4_ = H	[42]
Lucidenic acid H	R_1_ = OH	R_2_ = -OH	R_3_ = O	R_4_ = H	[44]
Lucidenic acid I	R_1_ = -OH	R_2_ = O	R_4_ = O	R_4_ = H	[44]
Lucidenic acid J	R_1_ = -OH	R_2_ = O	R_3_ = O	R_4_ = -H	[44]
Lucidenic acid O	R_1_ = -OH	R_2_ = -OH	R_3_ = -OH	R_4_ = -OH	[49]
Lucidenic acid R	R_1_ = -OH	R_2_ = O	R_3_ = O	R_4_ = OCOCH_3_	[51]

**Table 4:** Potential pharmacological effects of lucidenic acids and derivatives.

Lucidenic Acids and Derivatives	Potential Pharmacological Effects	References
Lucidenic acid A	Anti-cancer	[11,46,54,55,56,57,58,59]
	Anti-inflammatory	[27,50,60]
	Anti-viral	[50,60,61,62]
	Neuroprotective	[15]
	Anti-hyperlipidemic	[63]
	Treatment of frostbite	[64]
Lucidenic acid B	Anti-cancer	[11,55,57,58]
	Anti-inflammatory	[65]
	Antioxidant	[16]
	Anti-viral	[62]
Lucidenic acid C	Anti-cancer	[11,43,55,56,57,58]
	Anti-viral	[50,60,62]
Lucidenic acid D1	Anti-cancer	[12,66]
	Anti-inflammatory	[65]
Lucidenic acid D2	Anti-inflammatory	[60,65]
	Anti-viral	[50,60]
Lucidenic acid E1	Anti-inflammatory	[65]
Lucidenic acid E2	Anti-cancer	[59]
	Anti-inflammation	[60]
	Anti-hypercholesterolemia	[67]
	Anti-hyperglycemic	[16]
	Anti-viral	[50,60]
Lucidenic acid F	Anti-viral	[50,60]
Lucidenic acid H	Treatment of frostbite	[64]
Lucidenic acid I	Immunomodulatory	[14]
Lucidenic acid L	Anti-inflammation	[65]
Lucidenic acid N	Anti-cancer	[11,46,55,56,57,58,59]
	Anti-viral	[62]
	Neuroprotective	[15]
	Anti-hyperlipidemic	[68,69]
Lucidenic acid O	Anti-viral	[49]
Lucidenic acid P	Anti-inflammatory	[60]
	Anti-viral	[50,60]
Lucidenic acid Q	Anti-hyperglycemic	[16]
Lucidenic acid R	Anti-inflammatory	[51]
Methyl lucidenate A,	Anti-viral	[50,60]
Methyl lucidenic E2	Neuroprotective	[15]
	Anti-hyperlipidemic	[69]
	Anti-viral	[50,60]
	Immunomodulatory	[14]
Methyl lucidenate F	Anti-hyperlipidemic	[69]
Butyl lucidenate N	Anti-hyperlipidemic	[70]
20(21)-Dehydrolucidenic acid N	Ant-viral	[9]
	Immunomodulatory	[14]
20-Hydroxylucidenic acid N	Anti-viral	[9,50,60]
Methyl lucidenate Q	Anti-viral	[50,60]

## Data Availability

Not applicable.

## Sample Availability

Samples of the compounds are not available from the authors.
